# Supplementary material for: Comparative multiomics analysis of cell physiological state after culture in a basket bioreactor
Source: Sci Rep. 2022 Nov 23;12:20161. doi: 10.1038/s41598-022-24687-4 (PMC9686226; doi:10.1038/s41598-022-24687-4)
Supplement: Supplementary file 1 — Supplementary Information 1. [file 41598_2022_24687_MOESM1_ESM.zip › raw data/Proteomic raw data/6.KEGGenrichment/EG.vs.CG/DOWN/pathway/src/04610.html]

map04610


- K01343

- Down regulated proteins

A0A0D9RRB2(Description:Plasminogen activator;Gene:PLAT;log2FC:-1.8476)

- K03984

- Down regulated proteins

A0A0D9REV5(Description:SERPIN domain-containing protein;Gene:SERPINA1;log2FC:-1.4311)

- K17252

- Down regulated proteins

A0A0D9RTW6(Description:Clusterin;Gene:CLU;log2FC:-1.0684)

- K06462, K06464

- Down regulated proteins

A0A0D9QZZ6(Description:VWFA domain-containing protein;Gene:ITGAX;log2FC:-1.9952)

Close
